# Generation of genetically engineered CHO cell lines to support the production of a difficult to express therapeutic protein

**DOI:** 10.1186/1753-6561-7-S6-P1

**Published:** 2013-12-04

**Authors:** Holger Laux, Sandrine Romand, Anett Ritter, Mevion Oertli, Mara Fornaro, Thomas Jostock, Burkhard Wilms

**Affiliations:** 1 Novartis Development Integrated Biologic Profiling, 4002 Basel, Switzerland; 2 Novartis Institutes for Biomedical Research, 4056 Basel, Switzerland

## Introduction

Chinese Hamster Ovary (CHO) cells are widely used for the large scale production of recombinant biopharmaceuticals. These cells have been extensively characterised and approved by regulatory authorities for production of biopharmaceuticals. During the last years more and more cell-line engineering strategies have been developed to enhance productivity and quality. CHO cell line engineering work has made remarkable progress in optimizing products or titers by focusing on manipulating single genes and selecting clones with desirable traits. In this work it is shown how cell line engineering approaches enable the expression of a challenging to express "novel therapeutic protein". The expression of the "novel therapeutic protein" in CHO cells resulted in significant reduced cell growth as well as low productivity.

## Results

### Transcriptomics analysis

**Figure 1:**
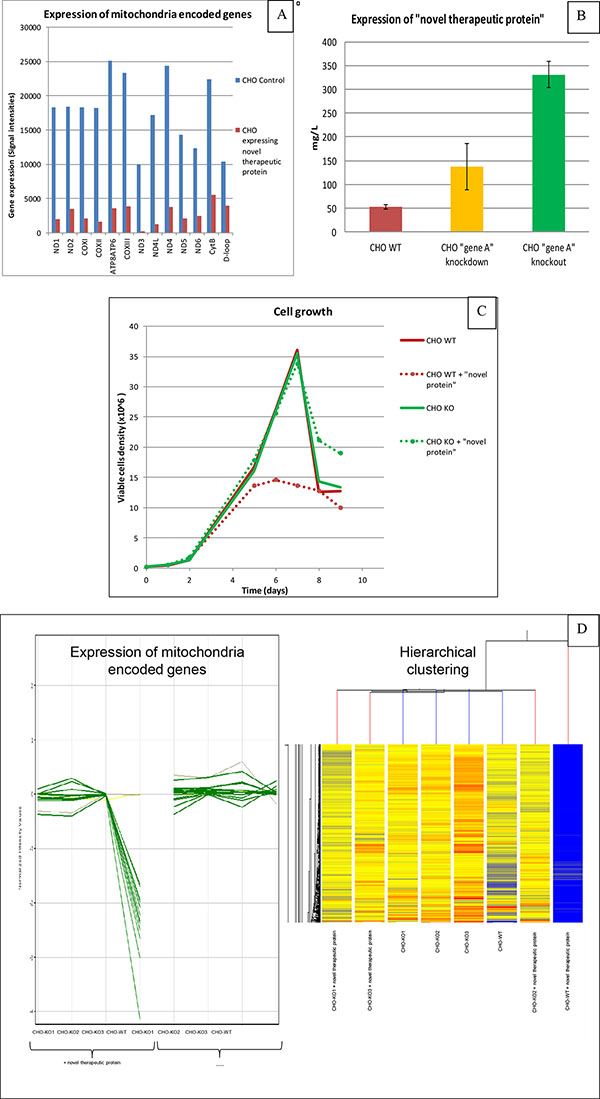
**A** highlights the reduced expression of mitochondria encoded genes in CHO cells expressing the "novel therapeutic protein" in comparison to parental CHO cells. The y-axis shows the gene expression values in signal intensities. **B**: Batch culture titers of the "novel therapeutic protein" in shake flask are shown. Titer for CHO WT cells are labeled in red, titer for CHO cells with reduced expression of gene A (shRNA approach) are labeled in yellow and titer for CHO cells with non-functional gene A (knockout) are labeled in green. **C: **Cell growth of CHO WT cells and CHO knockout (KO) cells with and without "novel therapeutic protein" in shake flasks. Y-axis shows viable cell density and x-axis cultivation time. **D: **Gene expression of mitochondrial encoded genes is not reduced in all three generated CHO KO cell lines with the "novel therapeutic protein". Hierarchical clustering reveals that the "novel therapeutic protein" does not affect the gene expression profile of the KO cell lines. In contrast the "novel therapeutic protein" has a clear effect on the WT cell line.

Using customised CHO specific microarrays the gene expression profile of CHO cells expressing the "novel therapeutic protein" was analysed. The expression of the "novel therapeutic protein" resulted in a significant downregulation of all mitochondria encoded genes. The downregulation was more than 40 fold for some of these genes (Figure 1A). This massive reduced transcription of mitochondrial encoded genes was very likely causing the reduced cell growth and reduced expression of the "novel therapeutic protein". A decrease in mitochondrial function reduces overall metabolic efficiency and a change of metabolic pathways could also be detected on gene expression level. Additionally the expression of "gene A" was detected in the applied CHO cell line, which might have the potential to trigger the down regulation of the mitochondrial encoded genes in the presence of the "novel therapeutic protein".

### Gene knockdown using shRNA (short hairpin RNA) technique

A variety of cell line engineering approaches were performed to circumvent cell growth inhibition caused by down regulation of mitochondrial encoded genes with the aim to improve expression of the "novel therapeutic protein". In the first approach the expression of "gene A", which was assumed to trigger the down regulation of the mitochondrial encoded genes, was repressed more than 10 fold using shRNA technique. shRNA is a sequence of RNA that makes a tight hairpin turn that can be used to silence target gene expression via RNA interference. Expression of shRNA was accomplished by delivery of stable integrated plasmids. Cells with reduced expression of "gene A" showed an improved cell growth and higher expression of the "novel therapeutic protein" (Figure 1 B). However cell growth was still repressed, although to a lower extent, and titers were still lower in comparison to other therapeutic protein formats. Despite the significant decrease in the expression of "gene A", the remaining "protein A" seemed to be sufficient to trigger these effects although to a lower magnitude.

### Gene knockout using zinc finger nucleases (ZFN)

To completely eliminate the cell growth inhibition a knockout of "gene A" was performed using ZFN technique. ZFNs are artificial restriction enzymes generated by fusing a zinc finger DNA-binding domain to a DNA-cleavage domain. Plasmids encoding ZFNs (specifically designed to detect and cleave "gene A") were transiently transfected in the parental CHO cell line. ZFN cleaves "gene A" which is then repaired by non-homologous end joining. This is often error prone and resulted in the generation of mutant alleles. Three clones were identified with mutation in both alleles of "gene A" resulting in shifts of the reading frame and therefore only nonfunctional premature termination products are encoded.

Knockout of "gene A" resulted in complete elimination of cell growth inhibition and the expression of mitochondria encoded genes (Figure 1D) was restored to levels comparable to parental CHO cells. In addition there was no change in the expression of genes that are involved in metabolic pathways. Most striking is the significant improved cell growth and productivity resulting in a 6-7 fold titer increase using this genetically engineered knockout cell line (Figure 1B and 1C).

## Conclusion

This example illustrates that transcriptomic analysis can support and facilitate the understanding and solving of specific issues during the expression of therapeutic proteins. Novel cell line engineering methods as ZFN technique are powerful tools to solve definite issues in production of therapeutic proteins in biopharmaceutical industry.

